# Genome size of 14 species of fireflies (Insecta, Coleoptera, Lampyridae)

**DOI:** 10.24272/j.issn.2095-8137.2017.078

**Published:** 2017-11-18

**Authors:** Gui-Chun Liu, Zhi-Wei Dong, Jin-Wu He, Ruo-Ping Zhao, Wen Wang, Xue-Yan Li

**Affiliations:** ^1^State Key Laboratory of Genetic Resources and Evolution, Kunming Institute of Zoology, Chinese Academy of Sciences, Kunming Yunnan 650223, China; ^2^University of Chinese Academy of Sciences, Beijing 100049, China; ^3^Center for Ecological and Environmental Sciences, Key Laboratory for Space Bioscience & Biotechnology, Northwestern Polytechnical University, Xi'an Shaanxi 710072, China

**Keywords:** Haploid genome size, Firefly, Flow cytometry, Evolution

## Abstract

Eukaryotic genome size data are important both as the basis for comparative research into genome evolution and as estimators of the cost and difficulty of genome sequencing programs for non-model organisms. In this study, the genome size of 14 species of fireflies (Lampyridae) (two genera in Lampyrinae, three genera in Luciolinae, and one genus in subfamily *incertae sedis*) were estimated by propidium iodide (PI)-based flow cytometry. The haploid genome sizes of Lampyridae ranged from 0. 42 to 1. 31 pg, a 3. 1-fold span. Genome sizes of the fireflies varied within the tested subfamilies and genera. *Lamprigera* and *Pyrocoelia* species had large and small genome sizes, respectively. No correlation was found between genome size and morphological traits such as body length, body width, eye width, and antennal length. Our data provide additional information on genome size estimation of the firefly family Lampyridae. Furthermore, this study will help clarify the cost and difficulty of genome sequencing programs for non-model organisms and will help promote studies on firefly genome evolution.

## INTRODUCTION

Fireflies, in the family Lampyridae (Coleoptera), are well-known as luminescent insects and include more than 2 000 species in approximately 100 genera of seven subfamilies worldwide (Branham, 2010; Lawrence & Newton, 1995). Different firefly species and their developmental stages exhibit different signaling systems, which play important roles in sexual communication and defense. As such, fireflies are a good model for studying the evolution of luminous signaling systems (Stanger-Hall & Lloyd, 2015; Stanger-Hall et al., 2007), sexual selection, and speciation (Lewis & Cratsley, 2008; Lloyd, 1971, 1973; Ohba, 1983).

Eukaryotic genomes not only contain genetic information but also act as structural components that determine nuclear properties and influence various biological features such as cell size, developmental rate, and developmental complexity (Gregory & Hebert, 1999; Koshikawa et al., 2008). Genome size is described by either mass (pg) or number of base pairs (bp) (Gregory, 2005a). Eukaryotic genome size is important as the basis for comparative research into genome evolution and as an estimator of the cost and difficulty of genome sequencing programs for non-model organisms (Gregory, 2005b; Gregory et al., 2007).

So far, the genome sizes of 5 635 animal species (3 793 vertebrates and 2 429 invertebrates) have been recorded in the Animal Genome Size Database (Accessed 27 March 2017) (Gregory, 2017). Compared to those of mammals (14.14%, 778 of 5 500 species) and birds (8.96%, 896 of 10 000 species), the genome sizes of invertebrates remain poorly studied regarding abundance and diversity. Of the nearly 1 000 000 described insect species, the genome sizes of only 930 (0.093%) have been estimated. Among them, more than two-thirds are from the Holometabolous orders Diptera (254 species), Coleoptera (181 species), Hymenoptera (153 species), and Lepidoptera (65 species) (Gregory, 2017). Coleoptera (beetles) (ca. 360 000 species) is the largest order in the animal kingdom (Bouchard et al., 2011, 2009), and its 181 species with reported genome size estimates are mainly distributed in nine families (Tenebrionidae: 69; Chrysomelidae: 65; Coccinellidae: 39; Dermestidae: 6; Scarabeidae: 3; Dytiscidae: 2; Carabidae: 1; Geotrupidae: 1; Silvanidae: 1). For the luminous beetle family (Lampyridae), the genome sizes of 23 species from North America have been described recently (Lower et al., 2017). Here, we report on genome size estimations of 14 firefly species from China.

To explore firefly genome size evolution and estimation of the cost and difficulty of future genome sequencing programs, we performed *C*-value measurements for 14 firefly species (two genera in Lampyrinae, three genera in Luciolinae, and one genera in subfamily *incertae sedis*) using flow cytometry. Although many methods for the estimation of genome size have been described, most genome size estimates in both animal and plant species estimations have been conducted using flow cytometry (Galbraith et al., 1983; Gregory et al., 2013; Hare & Johnston, 2011). We also constructed a phylogenetic tree of the 14 species using a mitochondrial cytochrome oxidase subunit 1 (*COI*) gene fragment and discussed firefly genome size evolution in the phylogenetic context. The relationships of genome size to morphological traits such as body length, body width, antennal length, and eye width were also described.

## MATERIALS AND METHODS

### Sampling and observation of morphological characteristics

Specimens of 14 firefly species from Yunnan, Hainan, and Hubei provinces of China were used for genome size estimation and body size measurement (Table 1). Some live specimens were used for estimation of genome size, with the remaining samples kept in 75% alcohol for morphological observation and body size measurement. All morphological observations and measurements were carried out under a dissecting microscope (SMZ 800, Nikon, Japan) according to Jeng et al.(2007). All measurements were based on male adults as females were difficult to collect. The abbreviations BL, BW, EL, ELW, PL, AL, and EYW represent body length, body width, elytral length, elytral width, pronotal length, antennal length, and eye width, respectively. BL is the sum of PL and EL (BL=PL+EL), BW is the greatest distance across the elytra, and EYW denotes the smallest interocular width (measured horizontally). Male genitalia were also dissected and examined under a dissecting microscope to help with specimen identification. According to previous morphological descriptions (Ballantyne et al., 2013; Jeng et al., 2000), all species were at least assigned to genus. For the four species with both male and female samples, live specimens collected at the same locality and time were observed to mate. Combined with their morphology, we confirmed they were of the same species.

**Table 1:** Sample information in this study

Family/subfamily	Species	*n*	Locality/Collection information
Lampyridae/*incertae sedis*	*Lamprigera yunnana*	20(8)	Kunming University of Science and Technology, Wuhua District, Kunming City, Yunnan, China, E102.694166°, N25.061163°, 7 September 2016, by Zhi-Wei Dong; Kunming Botanical Garden, Ciba Township, Wuhua District, Kunming City, Yunnan, China, E102.743100°, N25.138816°, 10 November 2007, Qing-Bai Hou et al, 21 September 2016, by Bao Wang et al; Jindian reservoir, Wuhua District, Kunming City, Yunnan, China, E102.776606°, N25.085929°, 21 September 2016, by Zhi-Wei Dong
	*Lamprigera sp1*	25	Xishuangbanna Tropical Botanical Garden, Mengla County, Xishuangbanna Prefecture, Yunnan, China, E101.269537°, N21.918722°, 8 November 2016, by Xue-Yan Li et al
	*Lamprigera sp2*	7	Nankang, Lujiang Township, Longyang District, Baoshan City, Yunnan, China, E98.768806°, N24.823003°, 8 November 2016, by Zhi-Wei Dong et al
Lampyridae/Lampyrinae	*Diaphanes nubilus*	12	Nankang, Lujiang Township, Longyang District, Baoshan City, Yunnan, China, E98.768806°, N24.823003°, 8 November 2016, by Zhi-Wei Dong et al; Dahaoping, Shangyun Township, Tengchong County, Baoshan City, Yunnan, China, E98.730027°, N24.976472°, 17 October 2003, by Xue-Yan Li et al
	*Diaphanes sp2*	12	Xishuangbanna Tropical Botanical Garden, Mengla County, Xishuangbanna Prefecture, Yunnan, China, E101.269537°, N21.918722°, 25 November 2016, by Zhi-Wei Dong et al
	*Diaphanes sp3*	12	Nankang, Lujiang Township, Longyang District, Baoshan City, Yunnan, China, E98.768806°, N24.823003°, 8 November 2016, by Zhi-Wei Dong et al
	*Pyrocoelia pygidialis*	16+2#	Kunming Botanical Garden, Ciba Township, Wuhua District, Kunming City, Yunnan, China, E102.743100°, N25.138816°, 20 August 2016, by Zhi-Wei Dong; Yuanjiang County, Yuxi City, Yunnan, China, 1986, by local villagers
	*Pyrocoelia sp1*	11	Datian, Dedang County, Lincang city, Yunnan, China, 8 October 2016, by Bo Ma
	*Pyrocoelia sp2*	15	Zongluzui Township, Tuanfen County, Wuhan City, Hubei, China 8 October 2016, by local villagers
	*Pyrocoelia sp3*	13	Baoshang, Miaoba village, Funing County, Wenshan Prefecture, Yunnan, China, 21 October 2006, by local villagers
Lampyridae/Luciolinae	*Abscondita terminalis*	50(20)	Menglun Township, Mengla County, Xishuangbanna Prefecture, Yunnan, China, 14 August 2015, by local villagers
	*Pygoluciola qingyu*	20(10)	Zhaotong City, Yunnan, China, 20 July 2003, by Hua-Li Chen
	*Pygoluciola sp1*	11(15)	Menglun Township, Mengla County, Xishuangbanna Prefecture, Yunnan, China, 11 August 2016, by local villagers
	*Luciola sp6*	20	Tunchang County, Hainan, China, 10 October 2016, by local villagers
*n*: Total number of males (females) per species. #: Larvae.			

For the males of each species, the brains of 3-6 live specimens were dissected for estimating genome size, with the thoraxes and abdomens were directly kept in -80 ℃ for genomic DNA extraction of single individuals when necessary. At least four males for each species were kept in 75% ethanol as voucher specimens. For females of the four species (*Lamprigera yunnana*, *Abscondita terminalis*, *Pygoluciola qingyu*, and *Pygoluciola sp1*), brains of 4-6 live specimens were dissected to use for estimating genome size.

### Flow cytometry

Genome size was estimated using flow cytometry (Bennett et al., 2003; Li et al., 2015). As with genome size estimation of other insects, such as the ladybird beetle (Gregory et al., 2003) and butterfly (Jiggins et al., 2005; Li et al., 2015), the model insect *Drosophila melanogaster* (genome size 176 Mb) (Bosco et al., 2007; Gregory & Johnston, 2008) was selected as the standard. Brain tissue from single firefly adults or larvae and the heads of 10 *Drosophila melanogaster* (*Dm*) adults were dissected under a dissecting microscope (SMZ 800, Nikon, Japan) and added to 60 μL of cold Galbraith buffer (Galbraith et al., 1983) in 1.5 mL Eppendorf tubes in Pestles (Sigma, USA) issue grinder, stroked 40 times with a pestle, and then added to cold Galbraith buffer to get a final volume of 400 μL for Lampyridae and 1 000 μL for *Dm*. Except for *Pyrocoelia pygidialis*, we prepared cell suspensions from 3-6 males and 4-6 females of Lampyridae as biological replicates. For *P. pygidialis*, only two larva individuals were used as biological replicates because no live adults were collected during the experimental period. Finally, the *Dm* and firefly cell suspensions were filtered through a 20 μm nylon filter. After this, 50 μL of the *Dm* cell suspension was added to 1.5 mL Eppendorf tubes containing 350 μL of the Lampyridae cell suspension. Propidium iodide was added to a final concentration of 50 parts per million, and the mixture was co-stained in the dark at 4 ℃ for 30-40 min. The fluorescence of co-stained nuclei for each sample was quantified using an LSR Fortessa (BD, USA) with the laser tuned at 561 nanometers. The DNA content (pg) was determined by comparing the ratio of the 2C mean of the tested samples with the 2C mean for *Dm* (1C=0.18 pg) (Bennett et al., 2003; Galbraith et al., 1983). Genome size (bp) was calculated from DNA content (pg) following the formula (Dolezel et al., 2003): genome size (bp)=(0.978×10^9^)×DNA content (pg). According to this formula, each *C*-value was calculated based on the main peak of the 2C cells.

### DNA extraction, PCR amplification, and sequencing

The genomic DNA of fireflies was obtained from the thorax and abdomen of a single male individual. DNA extractions were performed using a Gentra Puregene Blood Kit (Qiagen, Germany) following the manufacturer's protocols. The primers C1-J-2183 (5'-CAACATTTATTTTGATTTTTTGG-3') and TL2-J-3014 (5'-TCCAATGCACTAATCTGCCATATTA-3') (Lower et al., 2017; Simon et al., 1994) were used for amplification of the a part (about 800 bp) of the mitochondrial *COI* gene. The 20 μL reaction mixture consisted of 10 μL of 2×Trans Direct PCR SuperMix (Trans Direct Animal Tissue PCR Kit), 1 μL of forward primer (C1-J-2183) (10 μmol/L), 1 μL of reverse primer (TL2-J-3014) (10 μmol/L), 1 μL of DNA template, and 7 μL of ddH_2_O. The amplification protocol was as follows: initial denaturation and enzyme activation for 5 min at 94 ℃, followed by 35 cycles for 30 s at 95 ℃, 30 s at 55 ℃, 60 s at 72 ℃, with a final extension of 7 min at 72 ℃, and 10 ℃ hold. The PCR products were electrophoresed using 1% agarose gel and sequenced by BioSune BiotechnologyCo., Ltd (ShangHai, China).. The *COI* sequences of seven species were from our firefly mitogenome project (MG200080-MG200086); and those of the other seven species were from the current study and were deposited in GenBank under accession numbers (MF375910-MF375916).

### Phylogenetic analysis

All sequences were aligned using ClustalW and analyzed using MEGA 7.0 software (Kumar et al., 2016) and MrBayes version 3.1.2 (Huelsenbeck & Ronquist, 2001). Interspecific and intraspecific sequence divergences were calculated using the General Time Reversible (GTR+G+I) model with the pairwise deletion option in MEGA 7.0. Based on the GTR+G+I model, maximum likelihood (ML) tree was constructed using MEGA 7.0. Node supports for ML were inferred with bootstrap analysis (500 replicates). The Bayesian tree was established with MrBayes Version 3.1.2. The GTR+I+G model was selected via Modeltest version 3.7 and MCMC was run for 300 000 generations. The average standard deviation of split frequencies reached a value less than 0.01, with the Bayesian posterior probabilities calculated from the sample points after the MCMC algorithm started to converge (Zhan & Fu, 2011). *Rhagophthalmus lufengensis* and *Rhagophthalmus ohbai* (GenBank accession No. DQ888607.1 and AB267275.1, respectively) were used as outgroups (Li et al., 2007). We used molecular phylogeny to correct for nonindependence of related species (Felsenstein, 1985; Lower et al., 2017).

### Analysis of relationship between body size and genome size

Body size measurements, including BL, BW, AL, and EYW were determined based on 4-5 male individuals (Table 2). The relationships between genome size and body size were plotted using ggplot2 (Wickham, 2016). Phylogenetic generalized least squares (PGLS) in the R package nlme (Pinheiro et al., 2017) was used to analyze correlations between genome size and explanatory variables.

**Table 2:** Summary of the genome size (GS, in pg and Mb) of males of 14 firefly species and body size information, including body length (BL), body width (BW), antennal length (AL), and eye width (EYW)

Species	GS (pg)	GS (Mb)	BL (mm)	BW (mm)	AL (mm)	EYW (mm)	N1	N2	Accession No.
Subfamily *incertae sedis*									
*Lamprigera yunnana*	1.066±0.011	1 042.4±10.9	17.14±0.133	7.54±0.133	2.12±0.058	2.98±0.08	5	5	MG200082
*Lamprigera sp2*	1.133±0.004	1 107.7±4.1	17.75±0.25	7.625±0.11	2.45±0.029	3.225±0.111	3	4	MF375916
*Lamprigera sp1*	1.31±0.014	1 281.0±13.3	18.36±0.117	8.96±0.051	2.56±0.04	3.42±0.02	5	5	MF375915
Subfamily Lampyrinae									
*Diaphanes nubilus*	0.525±0.018	513.0±17.2	12.6±0.187	4.5±0.158	3.38±0.49	1.96±0.024	5	5	MG200080
*Diaphanes sp2*	1.007±0.022	984.9±21.5	10.6±0.43	3.78±0.08	5.86±0.22	1.16±0.04	6	5	MF375910
*Diaphanes sp3*	1.201±0.04	1 174.8±39.3	16.1±0.66	7.36±0.15	5.32±0.177	2.78±0.073	6	5	MF375911
*Pyrocoelia sp3*	0.421±0.004	411.6±10.8	20.04±0.163	10.58±0.296	8.64±0.103	2.04±0.024	6	5	MF375914
*Pyrocoelia sp2*	0.513±0.003	501.9±3.4	16.9±0.43	6.76±0.103	6.48±0.27	2.1±0.063	6	5	MF375913
Pyrocoelia pygidialis*	0.743±0.021	726.2±20.4	12.8±0.255	5.4±0.13	5.24±0.068	1.14±0.024	2	5	MG200081
*Pyrocoelia sp1*	0.754±0.021	737.2±20.9	22±0.707	8.54±0.37	8.17±0.068	2.08±0.037	5	5	MF375912
Subfamily Luciolinae									
*Abscondita terminalis*	0.503±0.01	491.5±8.9	10.84±0.144	4.2±0.138	4.46±0.051	2.26±0.024	5	5	MG200084
*Pygoluciola sp1*	0.744±0.024	728.0±23.5	9.82±0.037	3.84±0.068	3.18±0.066	1.64±0.024	5	5	MG200085
*Pygoluciola qingyu*	1.121±0.114	1 096.1±111.2	14±0.161	4.76±0.025	4.38±0.058	1.24±0.025	5	5	MG200086
*Luciola sp6*	1.288±0.015	1 259.2±14.5	6.32±0.111	2.48±0.086	2.24±0.081	1.66±0.024	6	5	MG200083
All values of genome size and body size are shown as mean±*SE* with the number of individuals used in genome size experiments (N1) and in body size measurement (N2); ^*^: For *Pyrocoelia pygidialis*, two live larva-stage individuals were used in the GS experiment, and five adult specimens collected in 1986 and kept in 75% ethanol were used in body size measurement. All sequences were deposited in GenBank.									

## RESULTS

### Firefly morphology

**Figure 1:**
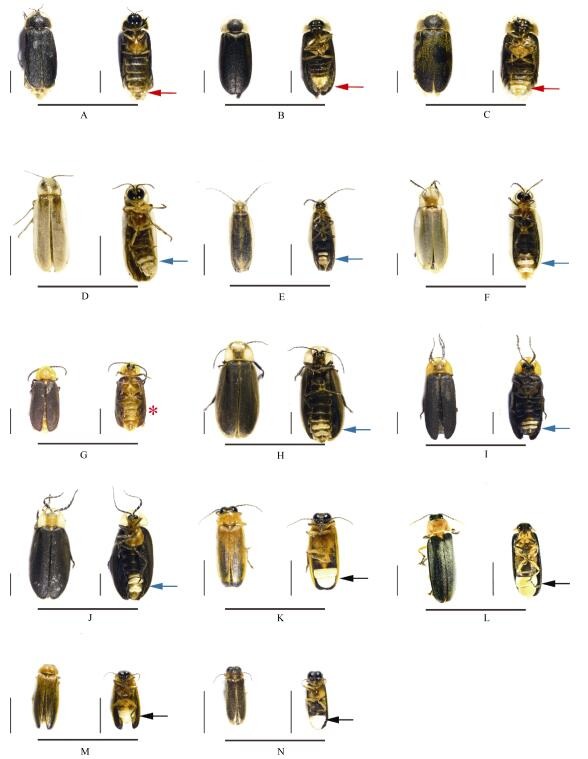
Habitus of 14 firefly species (All figures show dorsal view on the left and ventral on the right)

Considering that identification of fireflies at the species level is still unclear, especially for those species distributed in China, we assigned some specimens as species *incertae sedis* (*sp*) at a defined genus, and described their morphology (Figure 1, Table 2). *Lamprigera* was placed in the subfamily *incertae sedis* (Martin et al., 2017). Three species of *Lamprigera* had similar outer shapes (Figure 1A-C), but could be separated by their genital morphology. Three species of *Diaphanes* were easily separated by their antennae (Figure 1D-F). Four species of *Pyrocoelia* were separated by their wing and luminous organs (Figure 1G-J). Four species of Luciolinae were separated into three genera, including *Abscondita*, *Pygoluciola*, and *Luciola* by their wing, abdomen, luminous organs, and genitalia (Figure 1K-N).

### Firefly genome size and evolution

**Figure 2:**
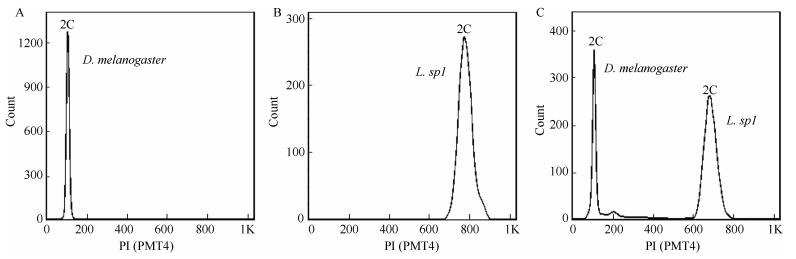
Number of nuclei measured by propidium iodide fluorescence PI(PMT4)-stained flow cytometry

Flow cytometry showed distinct peak(s) for the different species (Figure 2). Nuclei from the heads of the 10 *Dm* specimens and the brain of a single *Lamprigera sp3* male produced a single, broad 2C peak (Figure 2A-B), whereas mixtures of the heads of *D. melanogaster* and brain of the *Lamprigerasp1* male produced two broad 2C peaks (Figure 2C).

The haploid genome sizes of Lampyridae males ranged from 0.42 (*Pyrocoelia sp3*) to 1.31 pg (*Lamprigera sp1*) (411 Mb to 1 281 Mb) (Table 2), demonstrating 3.1-fold variation (Table 3). For four species: *Lamprigera yunnana, Abscondita terminalis, Pygoluciola qingyu, Pygoluciola sp1*, we also estimated the genome sizes of female individuals, which were found to be similar to those of the males (Table 4).

**Table 3:** Comparison of genome size for fireflies (Lampyridae) and other beetle families with described genome size

Family	Genera	Species	Genome size (pg)	Fold
Lampyridae (Asia)	6	14	0.42-1.31	3.1
(North America)	7	23	0.44-2.63	5.9
Carabidae	1	1	0.23	NA
Chrysomelidae	27	65	0.17-3.69	21.7
Coccinellidae	27	39	0.19-5.02	26
Dermestidae	1	6	0.90-1.98	2.2
Dytiscidae	2	2	1.01-1.22	1.2
Geotrupidae	1	1	0.83	NA
Scarabeidae	2	3	0.8-2.71	3.39
Silvanidae	1	1	0.25	NA
Tenebrionidae	28	69	0.16-0.87	5
NA: Not available because only one species was reported.				

**Table 4:** Summary of genome sizes (GS, in pg and Mb) of males and females from four firefly species

	Male		Female		N1 (Male)	N3 (Female)
**Species**	GS (pg)	GS (Mb)	GS (pg)	GS (Mb)		
Subfamily *incertae sedis*						
*Lamprigera yunnana*	1.066±0.011	1 042.4±10.9	1.051±0.033	1 028.1±32.6	5	6
Subfamily Luciolinae						
*Abscondita terminalis*	0.503±0.01	491.5±8.9	0.509±0.023	498.2±22.7	5	4
*Pygoluciola qingyu*	1.121±0.114	1 096.1±111.2	1.335±0.071	1 305.2±70.0	5	6
*Pygoluciola sp1*	0.744±0.024	728.0±23.5	0.758±0.03	741.5±29.2	5	6
All values of genome size are shown as mean±*SE* with number of males (N1) and females (N3).						

**Figure 3:**
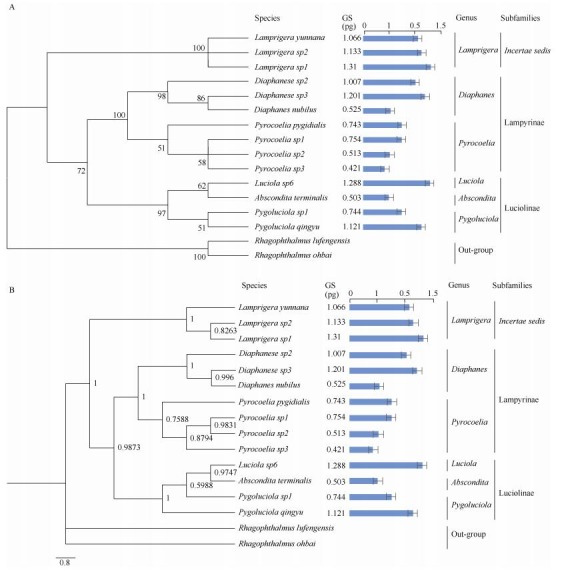
Phylogenetic trees of fireflies included in this study

To explore the evolution of genome size within Lampyridae, we constructed a molecular phylogenetic tree for the tested species using the mitochondrial *COI* sequences, which supported morphological taxonomy at the subfamily and genera levels (Table 1, Figure 3).

### Relationship between genome size and body size in fireflies

**Figure 4:**
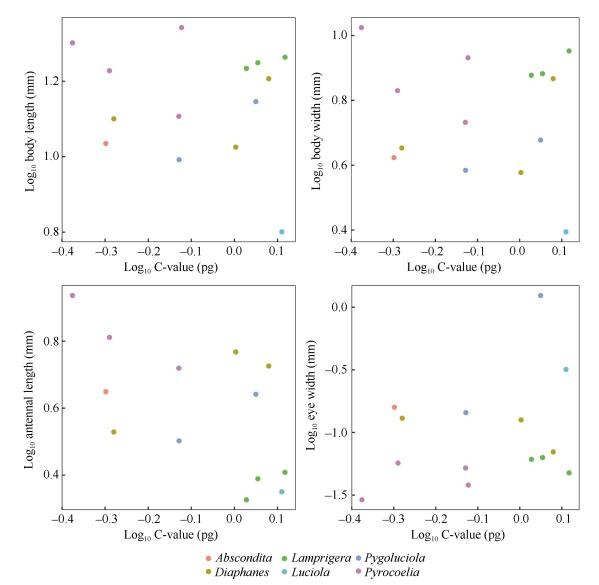
Relationships between diploid genome size and body size (mm) in fireflies

We explored the relationships between genome size and body size measurements, including BL, BW, AL, and EYW (Table 2). Our data showed no significant associations between firefly genome size and BL (*r*^2^=0.011, *P*=0.726, *λ*=1), BW (*r*^2^=0.016, *P*=0.669, *λ*=1), EYW (*r*^2^=0.11, *P*=0.241, *λ*=1), and AL (*r*^2^=0.045, *P*=0.469, *λ*=0.996) (Figure 4). We further performed PGLS analysis between BL, AL, EYW and phylogeny. The parameters of AL, EYW (*λ*=1), and BW (*λ*=0.996) indicated complete dependence on genome size between phylogeny and morphological traits. Pagel's parameter estimates for genome size supported a Brownian motion model of evolution and complete phylogenetic dependence (*λ*=1.00, 95%) supported a neutral model (Lower et al., 2017).

## DISCUSSION

Based on 39 species in 27 genera, the family Coccinellidae shows a large 26-fold genome variation (0.19-5.02 pg) (Table 3), with a considerable 21.7-fold variation also detected in Chrysomelidae (0.17-3.69 pg) according to 65 species in 27 genera (Gregory, 2017). A small 1.2-fold variation of genome size is reported in the family Dytiscidae (1.01-1.22 pg), though this is based on estimates of only two species. Our data from 14 species of six genera showed that the male haploid genome size in Lampyridae exhibited 3.1-fold variation (Table 3), which is relatively small compared to those of other currently estimated beetle families (Gregory, 2017) (Table 3). Nevertheless, compared to 2 000 species in more than 100 genera of seven subfamilies, the tested species in this study accounted for only a small proportion. Thus, more species, subfamilies, and genera, as well as different geographical distributions, are needed to better explore the evolution of firefly genomes. As Gregory (2002) states, the *C*-value enigma is a 'complex and multifaceted puzzle, immune to one dimensional explanations'.

Based on the phylogenetic relationship of the 14 species, our data suggest that genome sizes are very varied in Lampyridae. The *Lamprigera* species in subfamily *incertae sedis* exhibited a relatively large genome size of more than 1 pg (Table 2; Figure 3), which is less than 2-fold that of some *Pyrocoelia* species. The genome sizes of both Lampyrinae and Luciolinae ranged more than 2-fold. In Lampyrinae, *Pyrocoelia* species had relatively small genomes, spanning 0.42-0.75 pg (411-737 Mb), including the smallest known genome (0.42 pg, 411 Mb) in Lampyridae (Table 2); *Diaphanes* species showed relatively large genome size variation, spanning from 0.53-1.2 pg (513-1 174 Mb), in which *Diaphanes sp2* and *Diaphanes nubilus*, despite being closely related (Figure 3), showed 1.17-fold genome variation (Table 2). In Luciolinae, the genome sizes of *Pygoluciola sp1 and Pygoluciola qingyu* were 0.74 pg (728 Mb) and 1.21 pg (1 096 Mb), respectively; *Abscondita* *terminalis* had a relatively small genome (0.5 pg, 491 Mb), but related *Luciola* (*L. sp6*) species had a large genome (1.29 pg, 1 259 Mb) (Table 2; Figure 3).

Except for *Lamprigera yunnana*, three species in Luciolinae exhibited slightly larger genomes in females than in males. According to karyotypic analysis of species in the subfamilies Lampyrinae, Luciolinae, and Photurinae, Lampyridae frequently showed X0/XX karyotype sex determination, with males of X0 and females of XX (Dias et al., 2007), possibly explaining the slightly larger genome size in females than in males. Combined with the facts that the neoXY type was also reported from one species in Photurinae (*Bicellonycha lividipennis*) and the supernumerary chromosome found in some species of Lampyrinae (Dias et al., 2007) and that *Lamprigera* still has a disputable position at the subfamily level (Jeng et al., 2000; Li et al., 2006), it is too early to explain the slight differences in genome size detected between males and females of this genera. Further karyotypic analyses of these genera should help to settle this question.

Our data showed no significant association between the firefly genome size and morphological traits such as BL, BW, and EYW (Figure 4). Previous data also support no correlation between genome size and body size in the beetle family Coccinellidae (Gregory et al., 2003) and in North American species (Lower et al., 2017). However, for the *Pimelia* and *Phylan* genera in the beetle family Tenebrionidae, negative correlations between genome size and body size have been reported (Palmer & Petitpierre, 1996; Palmer et al., 2003). For other insects such as aphids (Finston et al., 1995; Gokhman et al., 2017) and mosquitos (Ferrari & Rai, 1989) and other invertebrates such as turbellarian flatworms (Finston et al., 1995) and copepods (Gregory et al., 2000), a positive relationship between body size and genome size has been described.

Although the study of animal genome size has been ongoing for more than half a century, there is still a need to estimate the genome sizes of more animal groups by flow cytometry and further explore the evolution of genome size. Fast though costly next-generation sequencing technology will provide a complementary role for genome surveys, including genome size and complexity (Li et al., 2015). In summary, our study provides an estimation of the cost and difficulty of genome sequencing programs for non-model organisms, and will help promote studies on firefly genome evolution.

## ACKNOWLEDGEMENTS

We would like to thank the anonymous colleagues and villagers for help in collecting the firefly specimens used in this study. We also thank Lei Chen and Wei Liu for their comments on this manuscript
